# Screening of iron deficiency anaemia in early childhood

**DOI:** 10.1186/s12887-021-02725-w

**Published:** 2021-09-08

**Authors:** Sophie Jullien

**Affiliations:** Barcelona Institute for Global Health, University of Barcelona, Barcelona, Spain

**Keywords:** Iron deficiency anaemia, Screening, Children

## Abstract

We looked at existing recommendations and supporting evidence on the effectiveness of universal screening of iron deficiency anaemia (IDA) in children under five years of age for improving growth, cognitive function, and psychomotor development. We assessed the accuracy of the screening tests for detecting IDA, the efficacy of existing treatment for children with IDA, and the potential harms associated with screening and treatment.

We conducted a literature search up to the 18th of August 2019 by using key terms and manual search in selected sources. We summarized the recommendations and the strength of the recommendation when and as reported by the authors. We summarized the main findings of systematic reviews with the certainty of the evidence as reported.

There is no suitable test for IDA screening that is non-invasive with high accuracy for detecting IDA and there is uncertainty whether IDA in children causes cognitive and psychomotor delays. There is a lack of evidence on the effects of routine screening for IDA in asymptomatic children under five years of age on growth, cognitive and psychomotor development outcomes.

Universal screening of IDA in children under five years of age is not recommended by most organisations such as the Spanish Association of Primary Care Paediatrics, the United Kingdom National Screening Committee, and the United States Preventive Services Task Force, but is recommended by the American Academy of Paediatrics. However, selective screening of IDA is recommended in infants and children with risk factors including prematurity, low birth weight, and dietary risk factors.

## Background

### Introduction

The World Health Organization (WHO) European Region is developing a new pocket book for primary health care for children and adolescents in Europe. This article is part of a series of reviews, which aim to summarize the existing recommendations and the most recent evidence on preventive interventions applied to children under five years of age to inform the WHO editorial group to make recommendations for health promotion in primary health care. In this article, we looked at the efficacy of universal screening of IDA in children under five years of age for improving growth, cognitive function, and psychomotor development.

### Why is iron deficiency anaemia important?

Iron is necessary for the production of haemoglobin. When iron stores are too low, usually due to dietary iron deficiency, the haemoglobin synthesis is impaired and results in iron deficiency anaemia (IDA). IDA is defined as haemoglobin level under two standard deviations for age and sex (< 11 g/dL for children between six months and five years), with serum ferritin under 10 to 12 μg/L [1, 2]. Children under five years of age are especially at risk, and the major concern is that IDA may affect cognitive and psychomotor development.

### Context

WHO estimated the worldwide prevalence of anaemia at 42.6% in children between 6 and 59 months of age in 2011 [3]. Focusing on the European region, it was estimated that 22.9% of children between 6 and 59 months had anaemia, translating to 12.7 millions of children with anaemia. Iron deficiency (ID) is the most prevalent nutritional deficiency in children and the major cause of anaemia. WHO estimated that between 44 and 65% of children with anaemia in the European region were amendable to iron supplementation [3]. Prevalence of IDA in children under five years of age was recently estimated at between 1 and 4% in the UK, the US, and other developed countries [2, 4].

Although IDA most commonly presents without symptoms, ID and IDA have been associated with cognitive impairment and neurodevelopmental delay at short and long term. However, this was based in observational studies with methodological flaws such as confounding with nutritional and socioeconomic factors, which make the cause-effect of the relationship difficult to establish.

The aim of screening IDA in children is the prompt identification and therefore early treatment of anaemia, which may improve health outcomes including growth, cognitive, psychomotor, and neurodevelopmental outcomes, mortality, and quality of life. Most infants with no risk factors such as prematurity or low birth weight are at low risk of iron deficiency during the first six months of life, due to iron stores from the perinatal period. This explains why screening programme usually focus on children from six months of age [3].

Criteria for a good screening programme that were adopted by the WHO are summarized in the core document [5].

## Key questions

1. How accurate are the screening tests for detecting IDA in children under five years of age?
2. Is treatment of IDA effective for improving growth, cognitive function, and psychomotor development?
3. What are the potential harms derived from treating IDA in children under five years of age?
4. Does population-based IDA screening and treatment in children under five years of age improve growth, cognitive function, and psychomotor development?
5. What are the potential harms of screening children under five years of age for IDA?

## Search methods and selected manuscripts

We described the search methods, data collection and data synthesis in the second paper of this supplement (Jullien S, Huss G, Weigel R. Supporting recommendations for childhood preventive interventions for primary health care: elaboration of evidence synthesis and lessons learnt. BMC Pediatrics; 2021. 10.1186/s12887-021-02638-8).

The search was conducted on the 18th of August 2019, by manual search and by using the search terms “iron deficiency anaemia” and “iron deficiency”. From the WHO, we included a guide for programme managers on IDA published in 2001, and a document published in 2007 assessing the iron status. We found recommendations and their supporting evidence from the United States Preventive Services Task Force (USPSTF) (2015), PrevInfad (2011) and the United Kingdom National Screening Committee (UK NSC) (2017). We included recommendations from the Centers of Disease Control and Prevention (CDC); although published in 1998, we did not find any update document addressing this topic. We also included the 2010 recommendations from the American Academy of Paediatrics (AAP). We found no eligible document from the National Institute for Health and Care Excellence (NICE).

The search in the Cochrane library by using the search terms ‘iron deficiency’ or ‘iron deficiency anaemia’ in titles, abstracts or keywords returned 33 reviews and six protocols. By screening the titles and abstracts, we included one review.

All the included manuscripts for revision in this article are displayed in Table 1.

**Table 1:** Included manuscripts for revision

Sources	Final selected manuscripts
**WHO**	• Iron deficiency anaemia. Assessment, prevention and control. A guide for managers. 2001 [3]
	• Assessing the iron status of populations; 2007 [6]
**USPSTF**	• Siu 2015 – Recommendations [7]
	• McDonagh 2015 – Evidence support and systematic review [8], with update literature review in 2018 [2]. Full systematic review is also available [9].
**PrevInfad**	• 2011 recommendations and supporting evidence [1]
**CDC**	• MMWR 1998 – Recommendations to prevent and control iron deficiency in the United States [10]
**NICE**	• None
**UK NSC**	• 2017 recommendations [11]
	• Screening for iron deficiency anaemia in children under 5 years. External review against programme appraisal criteria for the UK National Screening Committee 2017 [4]
	• Reply from the UK NSC public consultation (basis for the final recommendations) [12]
**AAP**	• 2010 recommendations [13]
**Cochrane Library**	• Wang 2013 - Iron therapy for improving psychomotor development and cognitive function in children under the age of three with iron deficiency anaemia (Review) [14]

**Abbreviations:** AAP: American Academy of Pediatrics; CDC: Centers for Disease Control and Prevention; NICE: National Institute for Health and Care Excellence; PrevInfad: PrevInfad workgroup from the Spanish Association of Primary Care Pediatrics; UK NSC: UK National Screening Committee; USPSTF: US Preventive Services Task Force; WHO: World Health Organization

## Existing recommendations

We summarized the existing recommendations and the strength of recommendations as per their authors in Table 2.

**Table 2:** Summary of existing recommendations

Source	Ref	Date	General recommendations for anaemia by iron deficiency screening
**WHO**	[3]	2001	For countries with adequate resource conditions, screening with haemoglobin or haematocrit and additional tests (serum ferritin or transferrin saturation) is recommended.
**USPSTF**	[7]	2015	‘The USPSTF concludes that the current evidence is insufficient to assess the balance of benefits and harms of screening for iron deficiency anemia in children ages 6 to 24 months.’ (I statement)
			‘This recommendation applies to children ages 6 to 24 months living in the United States who are asymptomatic for iron deficiency anemia. It does not apply to children younger than age 6 months or older than 24 months, children who are severely malnourished, children who were born prematurely or with low birth weight, or children who have symptoms of iron deficiency anemia.’
**PrevInfad**	[1]	2011	• Universal screening: it is recommended NOT to screen IDA (Low quality of the evidence, strong recommendation)
			• Screening in high-risk groups: IDA screening is recommended in all preterm infants under 1500 g of weight. ‘The benefits of routine screening, once the prophylaxis for IDA in asymptomatic premature children under 1500 g or less than 32 weeks is over, is greater than the potential damage.’ There is no evidence to recommend a second screening among children with risk factors who had a normal first screening. (Low quality evidence, weak recommendation)
**CDC**	[10]	1998	• Universal screening: “In populations of infants and preschool children at high risk for iron-deficiency anemia (e.g., children from low-income families, children eligible for the Special Supplemental Nutrition Program for Women, Infants, and Children, migrant children, or recently arrived refugee children), screen all children for anemia between ages 9 and 12 months, 6 months later, and annually from ages 2 to 5 years.”
			• Selective screening: “In populations of infants and preschool children not at high risk for iron-deficiency anemia, screen only those children who have known risk factors for the condition:
			° Consider anemia screening before age 6 months for preterm infants and low-birth weight infants who are not fed iron-fortified infant formula. ° Annually assess children aged 2–5 years for risk factors for iron-deficiency anemia (e.g., a low-iron diet, limited access to food because of poverty or neglect, or special health-care needs). Screen these children if they have any of these risk factors. ° At ages 9–12 months and 6 months later (at ages 15–18 months), assess infants and young children for risk factors for anemia […].”
**UK NSC**	[11]	2017	• “A ﻿systematic population screening programme for iron deficiency anaemia in children under 5 is not recommended.”
**AAP**	[13]	2010	• Universal screening: recommended at approximately 1 year of age with determination of haemoglobin concentration and an assessment of risk factors associated with ID or IDA.
			• Selective screening: recommended at any age in children who are at increased risk for ID or IDA.
			• If haemoglobin < 11 g/dL or if high risk of dietary ID, additional screening tests for evaluating ID/IDA with serum ferritin and C-reactive protein, or reticulocyte haemoglobin.
			• If mild anaemia (haemoglobin between 10 and 11 g/dL), close monitoring and document a 1 g/dL increase in plasma Hb concentration after 1 month of appropriate iron-replacement therapy.

**Abbreviations:** AAP: American Academy of Pediatrics; CDC: Centers for Disease Control and Prevention; ID: iron deficiency; IDA: iron deficiency anaemia; NICE: National Institute for Health and Care Excellence; PrevInfad: PrevInfad workgroup from the Spanish Association of Primary Care Pediatrics; UK NSC: UK National Screening Committee; USPSTF: US Preventive Services Task Force; WHO: World Health Organization

## Existing evidence

Two systematic reviews were recently commissioned by the USPSTF and the UK NSC to update their 2006 and 2012 recommendations respectively, based on the latest evidence available.

The USPSTF review included trials and control observational studies assessing screening in children between six and 24 months of age conducted in developed countries, as well as the effectiveness and harms of routine iron supplementation, and the association between a change in iron status and improvement in child health outcomes [8]. Studies conducted in resource-poor populations were excluded. Of note, the focus was on ‘studies that involved iron supplementation and treatment regimens commonly used in clinical practice in the US.’ The literature search was conducted up to August 2014 with the aim to identify new studies to update the previous USPSTF systematic reviews [15, 16]. Review authors identified ten new studies addressing routine iron supplementation (which is beyond the scope of this summary), and no new study addressing any of the key questions established on screening for IDA. An update of the search was conducted in June 2018 and identified one study, but this study did not address the key questions on IDA screening [2]. For each of the key question below, we therefore summarized the available evidence from studies that were identified from the previous USPSTF systematic reviews.

The UK NSC structured the review in four key questions to look at the evidence supporting IDA screening in children under five years of age [4]. The review authors included different types of studies for each key question, but only included studies with children from the UK or other similar population. The literature search was conducted up to March 2017.

### Effectiveness of population-based IDA screening in children under five years of age

We found no studies that evaluated the effectiveness of IDA screening in asymptomatic children between six and 24 months of age [8].

### Accuracy of IDA screening in asymptomatic children under five years of age

There is no single gold standard test to use as a screening tool for detection of IDA. Serum haemoglobin or haematocrit is usually the first test used to detect anaemia. However, this does not allow to distinguish between IDA and other causes of anaemia. Additional tests such as serum ferritin, transferrin saturation, erythrocyte protoporphyrin, and C-reactive protein (CRP) are required to detect iron deficiency.

Haemoglobin or haematocrit has a sensitivity estimated at 73% for detecting IDA, with a lower specificity estimated at 25%, as around half of cases with anaemia result from other causes than iron deficiency [1, 7]. The positive predictive value (PPV) of low haemoglobin for iron deficiency was estimated from 10 to 40% in children of 12 months of age [7]. PPV was found to be low if anaemia prevalence is under 10%, but increases in areas where anaemia prevalence is higher than 10% [1].

According to USPSTF review, there is a lack of data evaluating the sensitivity and specificity of other single tests for the detection of iron deficiency, such as serum ferritin, transferrin saturation, erythrocyte protoporphyrin, and CRP. Serum ferritin and CRP are acute phase reactants, and should therefore be measured in absence of infection or inflammation to avoid false positive results [7]. Serum ferritin is however considered as a ‘useful laboratory measure of iron status, with a low value being diagnostic of iron deficiency’ [9]. In 2007, the WHO and the CDC published a report on the assessment of the iron status in populations. This document was the result of a consultation that aimed to review and select the best indicators available to assess the iron status of populations and to evaluate the impact of interventions to control ID [6]. The authors reviewed the literature on indicators of iron status, ‘including red blood cell parameters, ferritin, free erythrocyte protoporphyrin, serum and plasma iron, total iron binding capacity, transferrin saturation and serum transferrin receptor’ and on the interpretation of indicators of iron status during an acute phase response. The authors reached the conclusions for recommendations that ‘measurements of serum ferritin and transferrin receptor provide the best approach to measuring the iron status of populations’, although ‘in places where infectious diseases are common, serum ferritin is not a useful indicator because inflammation leads to a rise in the concentration of serum ferritin as a result of the acute phase response to disease.’ Also, ‘serum ferritin is the best indicator of a response to an intervention to control iron deficiency and should be measured with the haemoglobin concentration in all programme evaluations’ [6]. Although this review and recommendations were focused to assess the iron status in populations, findings might be considered for application to IDA screening.

As an invasive blood test for population-based screening in children is unlikely to be accepted, the UK NSC review aimed to identify studies that would assess the diagnostic accuracy of non-invasive or minimally invasive screening test (such as urinary hepcidin) against a valid reference standard for identification of IDA (such as serum haemoglobin, haematocrit, serum ferritin or transferrin saturation levels) [4]. Review authors aimed to include studies that assessed screen test in a non-selected sample representative of the general UK population. However, no studies were identified that fulfilled their inclusion criteria.

A public consultation on IDA was conducted during the three months following the UK NSC publication of their updated recommendations in 2017 [12]. A German university replied to inform about a new non-invasive technique developed for the detection of erythrocyte zinc protoporphyrin by an optical measurement on the lower lip. According to this source, applying this technique to children under five years of age is feasible, and has a sensitivity of 83% and specificity of 93% against soluble transferrin receptor (for a threshold at 50 μmol / mol heme) [12].

### Potential harms of screening children under five years of age for IDA

Potential harms of screening include false positive results, leading to parents anxiety, and cost [7].

The 2015 USPSTF review found no studies that evaluated the direct harms derived from routine IDA screening on child health outcomes, and inadequate evidence on the harms of treatment of IDA in children between 6 and 24 months of age [8].

### Effectiveness of IDA treatment for improving health outcomes

#### Association between IDA and health outcomes

ID and IDA have been associated with cognitive and developmental delays, and some data showed that outcomes improve when children are identified and treated early. But this was from observational studies with methodological flaws and without an untreated control group [4].

None of the included systematic reviews identified any prospective study that would assess the effect of ID or IDA in children on developmental or other adverse health outcomes. No case control studies assessing clear historic data on ID or IDA status in children with adverse health outcomes were identified either [4, 8].

The UK NSC review concluded that ‘although iron treatment improves iron status in young children, there is conflicting evidence whether it has any effect of longer term health or developmental outcomes such as cognition’ [4].

#### Effectiveness of IDA treatment

Oral iron in different forms is usually used for treating IDA in children, but iron can also be administered parenterally.

A Cochrane review was conducted with the aim to determine the effects of iron therapy on psychomotor development and cognitive function among children under three years of age with IDA [14]. The review authors conducted the literature search up to April 2013 and included eight trials. The pooled difference in pre- to post-treatment change in Bayley Scale Psychomotor Development Index between iron and placebo groups was − 1.25 (95% CI − 4.56 to 2.06; 5 trials) and in Bayley Scale Mental Development Index was 1.04 (95% CI − 1.30 to 3.39; 5 trials), with a low quality of the evidence for both outcomes. The review authors concluded that there was ‘no convincing evidence that iron treatment of young children with IDA has an effect on psychomotor development or cognitive function within 30 days after commencement of therapy’ and that ‘the effect of longer-term treatment remains unclear.’

The 2015 USPSTF review found no new study that looked at efficacy of oral iron for treating IDA in children between six and 24 months [8]. Their previous systematic review included only one study published in 1986, which is included in the Cochrane review (described above). This study showed improved growth velocity as well as haemoglobin and ferritin levels, but no differences in psychomotor development. Authors of the USPSTF review judged this study as poor quality ‘due to baseline differences in age and unclear reporting of methods.’

The UK NSC review identified two systematic reviews [4]. The Cochrane review already described above, and one review published in 2013, that evaluated the effect of iron therapy on developmental outcomes in pre-school children with non-anaemic iron deficiency, which does not reply to the question of the effect of iron in treating IDA [14]. No further trials addressing this question were found.

### Potential harms of treating IDA in children under five years of age

Reported adverse effects of oral iron include ‘limited gastrointestinal symptoms, darkening colour of stool, staining of teeth and gums, and drugs interactions with other medications’ [7]. The Cochrane authors did not include adverse effects from iron treatment as an outcome in their review [14]. The 2015 USPSTF review reported findings from one randomized controlled trial published in 1991. From 334 children, no differences were found between the iron and placebo groups in overall incidence or incidence of specific adverse events, including gastrointestinal events [8].

## Summary of findings

- There is no suitable test for IDA screening that is non-invasive with high accuracy for detecting IDA.
- There is uncertainty whether IDA in children causes cognitive and psychomotor delays.
- There is a lack of evidence on the effects of treatment of IDA in children for improving growth, cognitive and psychomotor development outcomes.
- There is a lack of evidence on the effects of routine screening for IDA in asymptomatic children under five years of age on growth, cognitive and psychomotor development outcomes.
- Universal screening of IDA in children under five years of age is not recommended by most organisations such as PrevInfad workgroup (Spanish Association of Primary Care Paediatrics), the United Kingdom National Screening Committee, and the United States Preventive Services Task Force, but is recommended by the American Academy of Paediatrics.
- Selective screening of IDA is recommended in infants and children with risk factors including prematurity, low birth weight, and dietary risk factors (PrevInfad, United States Preventive Services Task Force, American Academy of Paediatrics).
- Well-designed controlled trials looking at benefits and harms of IDA screening in children for early diagnosis and treatment on short- and long-term health outcomes are needed.

## Data Availability

Not applicable.
